# Trajectories of Parental Daily Stress: An Ecological Momentary Assessment Study during the COVID-19 Lockdown

**DOI:** 10.3390/ijerph20116008

**Published:** 2023-05-31

**Authors:** Daniela Aldoney, Soledad Coo, Janet Carola Pérez, Andrés Muñoz-Najar, Constanza González, Manuel Montemurro, Leonel Tapia, Sofía Gana, Luz María Silva, Carolina Panesso, Jaime Silva

**Affiliations:** 1 Facultad de Psicología, Universidad del Desarrollo, Santiago 7610658, Chile; 2 Center for Social and Cognitive Neuroscience (CSCN), School of Psychology, Universidad Adolfo Ibáñez, Santiago 7550313, Chile

**Keywords:** stress, parenting, co-parenting, ecological momentary assessment, COVID-19 lockdown, parental education, socioeconomic status

## Abstract

The COVID-19 pandemic was a source of significant stress due to health and safety concerns and measures to control the virus’ spread, such as mobility restrictions. This measure was especially demanding for parents with school aged children, who had to find new work–family balance as their children participate in online education while attempting to work remotely. To evaluate parents’ stress trajectories during the pandemic, we conducted Ecological Momentary Assessments (EMAs) during lockdown for 29 days in 68 families in Santiago, Chile. In addition, we evaluated the role of educational level and income, co-parenting, and number of children in parents’ stress trajectories. Our results showed that during the first weeks of lockdown expected protective factors (i.e., income and co-parental support) were not able to influence parents’ daily stress management. Moreover, parents with higher educational levels reported worse stress adaptation than less educated parents. On the other hand, co-parental conflict was significantly associated with parent’s stress. Our study captured an acute response to COVID-19 related challenges. This study contributes to understanding how parents adjust to stress during adverse circumstances such as the COVID-19 pandemic.

## 1. Introduction

In 2020, people around the world had to deal with several stressors related to the COVID-19 pandemic. It triggered intense worries and fears about the physical health of self and others. The novelty of the situation and the lack of accurate information regarding the consequences and duration of the pandemic exposed families to elevated and prolonged periods of stress. This is concerning given the negative consequences of experiencing chronically elevated stress on well-being. In fact, COVID-19 has triggered mental health problems in various countries, increasing the prevalence of anxiety and depression, especially in young adults [1].

Many countries—as well as Chile—established social distancing and lockdown measures to control the virus outbreak. These included the closing of schools, universities, parks, and non-essential businesses, limiting movement and transportation, and promoting physical separation referred to as social distancing in order to control the spread of the virus.

During the lockdown phase, many adults were not permitted to go to work, and others had to transition to working remotely from home. The closure of childcare, schools, and many services meant an additional challenge for families with young children. Parents had to manage home schooling and childcare together with novel work conditions. The demands of maintaining this balance have been identified as one substantial source of stress [2,3].

Though parents’ increased stress levels during the pandemic have been well documented [4,5], less information is available about parents’ stress trajectory during the lockdown period and their ability to adapt to this trying situation. Effective stress management is essential for mitigating the deleterious impact of COVID-19, but individuals within the population vary greatly in their vulnerability to stressful life events [6]. Understanding individual and/or family dynamics that may alter parents’ stress trajectories is required in order to identify groups of parents who are more susceptible to negative pandemic-related outcomes and are thus in most need of support. Furthermore, such an analysis may also help identify factors that enable a better adaptation to the new circumstances.

The aim of this study is to examine the trajectory of perceived stress in a group of Chilean parents with young children during the first COVID-19- related lockdown. To do so, parents reported on their stress level every other day for 29 days. In addition, we investigated whether personal and familial variables predicted an increase or decrease in stress levels. 

### 1.1. Theoretical Background

The Transactional Model of Stress and Coping proposed by Lazarus and Folkman [7] suggests that an individual’s capacity to cope and adjust to challenging situations is a result of transactions (or interactions) that occur between the individual and the environment. According to this model, when confronted with external or internal circumstances, individuals engage in an appraisal process to assess the significance of the particular situation and their own ability to influence and/or cope with it to minimize any potential negative consequences. The stress response is triggered when individuals deem their personal resources and abilities insufficient to effectively deal with the event they are facing. Thus, the stressful character of a situation is more related to the significance the situation has for the individual than to its objective characteristics [8].

This model is consistent with the United Nations’ definition of a disaster [9] as one that entails the significant disruption of a community that involves diverse losses that exceed the community´s ability and resources to cope with it. Considering the wide range of human, economic, and material losses related to the COVID-19 pandemic as well as its disruption to the usual functioning of human groups, it can certainly be defined as a disaster.

From a complementary perspective, Hobfoll’s Conservation of Resources Theory [10] suggests that individuals are motivated to obtain and preserve what they value, which are considered resources. These involve a wide range of tangible and intangible items, from primary needs such as housing and sustenance, secondary resources such as social support and employment, and tertiary socially constructed resources like social status and accomplishment. When these resources are threatened or depleted, stress is triggered along with initiatives to protect existing resources and acquire new ones. The severity and duration of the stress response is associated with the magnitude of the resource loss. Further, resources are often correlated and nested within families and communities, which explains why some groups are more vulnerable to stress than others. By focusing on resources and resource loss, this model emphasizes the objective characteristics more than the subjective perception of a situation. The COVID-19 pandemic triggered a cascade of resource loss at both individual and community levels, which may explain its widespread stressful nature.

### 1.2. COVID-19 as a Stressful Event for Parents

As a response to the COVID-19 pandemic and to prevent it from growing, Chilean authorities imposed drastic measures in mid-March 2020, including restrictive lockdowns, a national curfew, business closings, and the complete shutdown of the educational system (i.e., daycares, schools, and universities) [11].

Only a few days after the school year started on March 16th, 2020, the Chilean government ordered a nationwide two-week school shutdown. This measure was extended for two more weeks and the winter vacation period—normally taken in July—was moved ahead to April.

**Figure 1:**
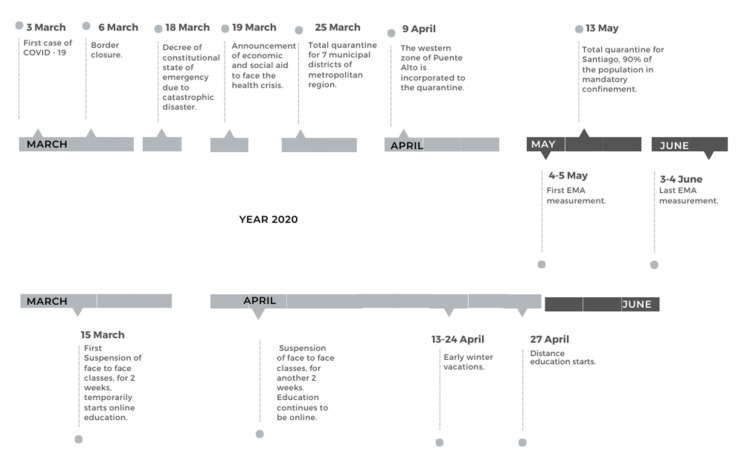
Timeline of the COVID-19 pandemic and mitigation actions in Chile (March–June 2020).

Schools had to switch to online education. The implementation of this modality revealed significant socioeconomic status (SES) gaps in students´ access to technology and the internet, large differences in teachers’ qualifications and schools´ practices for implementing online learning, and difficulties reported by parents in guiding their children through it [12,13,14,15]. In fact, most schools were closed for the duration of the first semester, and by the end of 2020 only one in ten schools had returned to in-person education [16] (see Figure 1).

As schools and childcare centers remained closed and as children participated in online education, parents had to take on new and unfamiliar roles and responsibilities [16,17]. Many parents often struggled with understanding the role they should play in their children’s online learning [16,17], resulting in heightened levels of stress [18].

By the beginning of May, almost the entire Metropolitan District was placed under strict lockdown. These measures lasted from three to four months and included different areas of Santiago (i.e., the capital city) and some urban areas in the rest of the country.

During the COVID-19 lockdowns, many companies and institutions had to introduce remote work, which forced people into new routines and interaction patterns. This was especially demanding for parents with school-aged children because they had to achieve a new work–family balance with their children attending online classes while attempting to work remotely (or were unable to work) and do household tasks with no clarity on how long the shutdown would last [18]. In Chile, the possibility of working remotely was more common for highly educated groups, those living in the Metropolitan District, and women [19]. In this regard, both national and international evidence show that the challenge of balancing the demands of the workplace with an increased domestic and caregiving workload was particularly marked for mothers [20,21].

During the pandemic, the general Chilean population showed an increase in feelings of fear, anxiety, and sleep difficulties [22]. In addition, the long periods of mobility restrictions and lockdown occasioned the separation from loved ones, the loss of freedom, and uncertainty over disease status and boredom, all of which had negative consequences on people’s mental health [23]. Parents of young children also experienced challenges with continuing to parent in the same way as they had prior to COVID-19, reporting that parenting had grown harder over the course of the pandemic. The most common factors that influenced parenting were changes in children’s daily structure and routines, worry and anxiety around COVID-19, and demands related to children’s online schooling at home [24]. A Chilean study with more than 6000 caregivers reported that parents’ concerns were connected to their children’s school-related work and well-being as well as finding time for themselves [25].

A smaller study with 719 parents showed the number of days in lockdown and their perceptions of children´s demands for parental attention increased caregivers’ risks of suffering parental burnout [26]. Another study reported an increase in symptoms of depression from the period just before the onset of the pandemic to the start of the lockdown, with these symptoms remaining high later on during the pandemic [27].

How parents and families manage stress is essential for mitigating the deleterious impact of COVID-19 on their own and their children’s mental health. Learning about individual and context-specific characteristics is essential for understanding which ones constitute risk factors for mental health problems and negative family interactions.

### 1.3. Individual Differences in Stress Adaptation

Individuals within the population vary greatly in their vulnerability to stressful life events [6,28]. A predisposition to maladjustment may arise in a variety of ways and at different stages of the life cycle. To develop targeted preventions and interventions aimed at the most vulnerable, we need to examine which individual and family characteristics influence adaptive responses to the COVID-19 pandemic.

As proposed in the theoretical framework, adaptive capacity to stress may depend on the resources that are threatened or depleted by a new situation, the lockdown in this case.

Research reveals several factors predicting differences in individual responses to stressful contexts like the COVID-19 pandemic. For example, higher SES (i.e., high education and/or income levels) parents may have more economic and individual resources to successfully manage the new family life organization [29,30]. Low-educated parents may also feel less able to assist their children during the online class period, which is an additional burden for parents [31].

A higher number of children elevates demands on parents and requires more time and energy from the caregiver [32]. This might diminish a parent’s ability to deal with added stressors, such as those experienced as a result of the COVID-19 pandemic [32,33]. Another aspect that diminishes parental capability to deal with stress is depression. Depression includes changes at the emotional, cognitive, and/or motor levels that affect interpersonal functioning. It is not uncommon for stress and depression to co-occur [34] without a clear understanding of the specific connection between the two [35].

Given the systemic aspects of parenting and family relations, the quality of co-parenting relationships may also play a role as a protective or risk factor for the negative impacts of the pandemic on parents. Co-parenting refers to two or more adults in any family structure engaging in the shared activities and responsibilities of raising a child [36,37]. Researchers have repeatedly found co-parenting to be a predictor of parenting quality and family stability [38,39,40]. A recent study showed a positive relationship between co-parenting and lower levels of COVID-19 related stressors. This relationship was especially significant for mothers from low socioeconomic backgrounds, suggesting that positive co-parenting could play a protective role against perceived stressors related to COVID-19 [40]. In contrast, higher levels of stress have been associated with lower-quality co-parenting [41]. A study in the Netherlands showed that greater increases in parental stress were associated with greater decreases in co-parenting quality at the height of the COVID-19 lockdown [42].

### 1.4. Present Study

Although various studies have investigated the effects of the COVID-19 pandemic on people’s mental health and while some have reported the consequences specifically on parents, little is known about the adaptation trajectories of this group to the great stressor that was the pandemic. It is important to bear in mind that this crisis was not an isolated event that erupted abruptly as other phenomena such as natural disasters or other tragedies can; rather, it was a dynamic process involving a wide range of changes, events, and uncertainty that lasted for several months. For this reason, it is important to understand the adaptation processes in terms of trajectories, identifying the factors that contributed to a better or worse adaptation to the ongoing crisis. This will enable the identification of people who are more susceptible to maintaining high stress levels and are therefore more in need of support interventions.

Based on the above information, we hypothesized that parents of families with higher education or income would report lower stress levels at the first assessment and/or better adjustment in their stress trajectory (i.e., lower or no increase in their stress levels across assessments) in comparison to parents with less education or income.

In addition, when considering the number of children living in the household, small families (parents with one child) would report lower stress at baseline and/or a better adjustment trajectory than families with two or more children. Finally, considering the range of events and uncertainty dynamics that characterized the COVID-19 pandemic, family variables were measured as time-varying predictors. Consistent with this methodological decision, our hypotheses are that greater co-parenting support would be related to lower daily stress in parents and that greater co-parenting conflict would be related to higher levels of daily stress in parents.

Considering that women are more likely than men to meet the criteria for an anxiety disorder [43,44] and perceived stress [45], being a mother was included as a control variable. Additionally, stressful life events have been shown to increase the risk of anxiety [46,47] and heightened distress [48], so recent stressful events were included as control variables. Additionally, given that anxiety and depressive symptoms are highly comorbid in both clinical [49] and community samples [50] and given that our previous study indicated that personality factors, specifically dependency and self-criticism, were positively correlated to parental depressive symptoms at the start of the lockdown [27], dependency, self-criticism, and previous parental depression were also included as control variables.

## 2. Materials and Methods

### 2.1. Sample

This Ecological Momentary Assessment (EMA) study involved a sub-sample of 68 families who had participated in a larger study before the COVID-19 pandemic. The original sample was recruited in 2018 and included 120 low- to middle-income Chilean families with cohabiting parents of a preschool child. This was a convenience sample. Among the children, 53% were girls, and 51.3% were in childcare. The mean age of mothers and fathers was 31.15 years (SD = 6.10) and 33.9 years (SD = 7.09), respectively. In terms of education, the majority of the mothers (87%) and fathers (86%) had a high school diploma or higher education.

We were able to reach and invite 64% (N = 77) of the families from the original sample to join this study, and 88% of them agreed to participate (N = 68 families). Eligibility criteria were the same as for the original sample: (1) adults (18 years old or older), (2) cohabiting parents, and (3) having a 5- or 6-year-old child. Parental exclusion criteria included having a diagnosis of intellectual impairment or a neurodevelopmental or severe psychiatric disorder (i.e., schizophrenia, bipolar disorder, major depressive disorder). Children’s exclusion criteria involved having a diagnosis of intellectual disability or a neurodevelopmental disorder.

### 2.2. Procedure and Data Collection

We recruited the original sample at four primary healthcare centers in southern Santiago, Chile’s capital city. Families were approached by research assistants in the waiting room, and posters invited parents of preschool children (aged 2.5 to 3.5 years) to join a study on play, learning, and development, which presented the research team’s contact details. We contacted the families by phone to invite them to participate in the present EMA study. Families who agreed to join the study received the participant information over the phone and got to ask any questions they may have had. The study’s information sheet and informed consent were also sent to them via an instant messaging application (WhatsApp, WhatsApp LLC; Meta Platform, Inc., version 2.19, Menlo Park, CA, USA) or e-mail.

The parents who agreed to participate completed EMA surveys measuring parent-child dynamics, parental stress, and co-parenting every other day for 15 days. This assessment began 21 days after the onset of the COVID-19 lockdown. Parents received one questionnaire with several scales to be completed during the day between 9:00 and 13:00 hours. via WhatsApp. We used an online platform (SurveyMonkey, Nomentive, version 2.1.09, Menlo Park, CA, USA) to design and administer the questionnaire. All participants had compatible mobile devices. Each questionnaire scale took up one page on the screen, and the final questionnaire was five pages in total.

Compliance was moderate. For the first measurement point, 72% of parents completed the survey. During the following 14 assessment time points, compliance ranged between 51% and 72%, with day number 25 (i.e., time point number 13) presenting the lowest compliance. On the final assessment day, 59% of parents had completed the study survey.

All participants received USD 7 upon completion. This study was approved by the University Institutional Review Board.

### 2.3. Variables and Instruments

Sociodemographic information. A brief set of questions asked about the participants’ age, number of children, highest educational level attained, and income. Both educational level and income were measured as categorical variables. Educational level categories included finishing primary school (or not), secondary school (or not), and college or higher education (completed or not). Monthly income categories were earning less than USD 250, USD 250 to 380, USD 380 to 640, USD 640 to 1300, and above USD 1300. An additional question asked about the number of stressful events that occurred between 2018 and April of 2020 from a list of ten possible events (e.g., accidents, unemployment, death of relatives, etc.). Finally, questions about COVID-19 information were included: (a) Have you been tested for COVID-19? (Possible response: No; yes, but I did not have the disease; yes, I had a confirmed case of COVID-19); (b) Have you or any relative been diagnosed with COVID-19? (Possible response: Yes/No). If yes, were you in the hospital? or Was hospitalization not required?

DASS-21/Stress subscale (DASS-21) [51]. The seven items of the stress subscale were used to examine the daily stress experienced by participants. The original instrument statements were shortened to fit mobile device screens, and their instructions were modified to allow every other day variability. Participants were asked, “During the last two days, think about how often you felt these emotions or the following situations happened to you.” Participants answered the items on a 4-point Likert scale (1 = “Rarely or at no time”, 4 = “Most or all of the time”). The DASS-21 has been adapted for use in Chile and has good psychometric properties with Cronbach’s alpha ranging from 0.79 [52] to 0.83 [53]. Cronbach’s alpha in this study ranged from 0.79 to 0.94.

Depression Scale of the Center for Epidemiological Studies (CES-D) [54,55]. This self-report questionnaire has ten items that assess depressive symptoms over the past week. The total score ranges from 0 to 30 with higher scores showing greater symptom severity. Scores higher than 10 indicate a risk of presenting a depressive disorder. This scale was applied in 2018. The Cronbach’s alpha was 0.82 and 0.76 for maternal and paternal reports, respectively.

The Co-parenting Relationship Scale [56] is a 35-item scale with seven subscales: Co-parenting Agreement, Co-parenting Closeness, Exposure to Conflict, Co-parenting Support, Co-parenting Undermining, Endorse Partner Parenting, and Division of Labor. In this study, only two subscales were applied: Exposure to Conflict (5 items, for example “Do you argue with your partner about your child in the child’s presence?”) and Co-parenting Support (6 items, for example “My partner appreciates how hard I work at being a good parent”). The first-dimension accounts for the degree to which parents experience conflict in their relationship, and the latter assesses parents´ perception of co-parental support from their partner. The instructions and response scale were modified to assess every other day variability. Participants were asked to “Answer as honestly as possible, thinking about the last two days”. The items had a 4-point Likert scale (1 = “Rarely or at no time”, 4 = “Most or all of the time”). Items were translated and back translated to Spanish by bilingual members of the research team. The results of an exploratory factor analysis performed for the first measurement time support a two-factor solution for these 11 items. In this solution, items were loaded on their respective Exposure to Conflict and Co-parenting Support factors, which were correlated (r = −0.44). Cronbach’s alpha in this study ranged from 0.78 to 0.95 for the Conflict subscale and from 0.86 to 0.96 for the Co-parenting Support subscale.

The Depressive Experiences Questionnaire (DEQ; [57]) is a self-report, 66-item questionnaire based on the polarities of experience model [58]. It has been widely used in personality and character styles research [59] and addresses dependency and self-criticism. Dependency measures core characteristics of an anaclitic depression such as loneliness, helplessness, and fear of rejection. Self-criticism describes a self-critical or introjective depression with feelings of worthlessness, inadequacy, guilt, and critical self-monitoring. The scores for these dimensions range from −3.5 to +3.5 points (standardized score) and are obtained following scoring instructions, which consider all the items, but with different weights [60]. Therefore, the reliability of the scale as a whole was calculated using Cronbach’s alpha (value 0.87). Participants were asked to complete this questionnaire one week after the end of the EMA surveys).

### 2.4. Data Analysis

We conducted a descriptive analysis of the data at each of the assessment times and estimated Pearson correlations between time-varying variables. Additionally, we analyzed time invariant predictors, wherein mothers’ and fathers’ ratings were compared by dependent-samples *t*-tests. To account for the variability in the level of parents’ stress during the 29 days of the first full COVID-19 pandemic lockdown, a Two-Level Hierarchical Linear Model was modeled using a linear mixed model. The analyses were conducted with IBM-SPSS v.23 (IBM Corp., Version 23.0., Armonk, NY, USA), REML estimation method, using a model-building approach [61].

From a theoretical perspective, the parents’ stress could be included into a three-level model. Alternate day assessments (Level 1) were nested within persons (Level 2) and then nested within families (Level 3). It is reasonable to propose that mothers and fathers belonging to the same family have some degree of data non-independence based on their kinship linkage or their daily interactions [62]. Nevertheless, this assumption must be evaluated empirically. Using the ML−2∆LL test [61], a Single-Level Model (independent data) was compared with a Two-Level Model (time points nested within persons). The latter was compared to a Three-Level Model (time points nested within persons, and both nested within families), showing that a Two-Level Model was more adequate than a Single-Level Model; ML−2∆LL test = 970.7 (1 df.), *p* < 0.001. Nevertheless, the Three-level Model fit did not improve with respect to the previous one, ML−2∆LL test = 1.9 (1 df.), *p* = 0.173, indicating that there was no between-family intercept variation. Thus, a Two-Level Model composed of time points nested within persons was created.

First, the Null Model (Model 1) was estimated to account for the intraclass correlation (ICC). We estimated Model 2 (Random Linear) and Model 3 (Fixed Quadratic) to account for the evolution of participants’ stress levels during the initial COVID-19 pandemic lockdown. A time variable was estimated as a numeric sequence from 0 to 14, capturing each of the EMA assessments conducted every other day. By doing this, the intercept shows mothers‘ and fathers’ stress levels on the first assessment day. In Model 4, Co-parental Support and Exposure to Conflict were included as random predictors of participants’ stress. In order to separately represent between-person and within-person variation, we estimated two new predictors of each one of these variables. For example, in the case of Co-parental Support, the within variable (Level-1) was estimated using person-mean-centered, and a between variable (Level-2) was estimated using grand mean-centered of person-mean of the time varying co-parental support variable across days. This procedure could capture differences within a person (e.g., some days mothers or fathers report higher stress than they usually report) and between persons (e.g., some persons report more stress than others).

To account for the impact of education, family income, and number of children per household on the trajectory of participants’ stress levels during the initial COVID-19 lockdown, we included these variables as predictors of the intercept and the linear slope (Model 7). Based on variable frequencies, a series of dummy variables was created. The number of children was operationalized using two dummy variables, namely two children and three or more children, with “one child” as the reference group. Considering that 12 years of combined primary and high school education are mandatory in Chile [63], educational level was converted to a dummy variable according to parents’ education being over 12 years (coded as 1) or not (coded as 0). Income was coded into two dummy variables: USD 380 to 1300 and higher than USD 1300 (which represents the 16% richest Chilean population [64]) with less than USD 380 used as the reference group. The latter corresponds to Chilean minimum wage at the point of March 2019 [65]. The variables previous depression, being a mother, stressful events, and personality dimensions (i.e., dependency and self-criticism) were included as control variables. All the control variables were grand-mean centered except “being a mother”.

Finally, conceptualizing a current COVID-19 diagnosis within the family as a relevant stressor, the final model (Model 7) was re-estimated including a COVID-19 diagnosis in the parents and their families as dummy predictors (COVID-19 Diagnosis in Parents or/and their relatives and COVID-19 Exam). The results indicated that the added predictors were not associated with the stress reported by parents (i.e., non-significant parameters). These results are available as Supplementary Material.

## 3. Results

### 3.1. Characteristics of the Sample

Participant’s reports showed that 80.9% of the mothers lived with their partner (also the child’s other parent), and the majority of them (98.5%) lived with their child/children all of the time, with only 1.5% of mothers living with their child/children some days per week. When asked about the number of children per household, 26.5% of the mothers reported having a single child; 33.8% reported having two; and 39.7% had three or more children. With regard to the highest maternal educational level achieved, 35.3% had 12 or less years of education; 36.8% did not finish higher education; and 25% had completed college or higher education.

Chile guarantees 12 years of compulsory education, but the majority of the adult population (88%) holds a high school diploma. The mean year of completed education is 11.05. In this study, families are considered low- or middle-income if they have an average monthly household income between USD 734 and 1468 [66].

At the beginning of the study, four mothers (5.9%) and two fathers (3.2%) reported having had the diagnostic test for COVID-19, but the diagnosis was confirmed in only one mother and one father. Additionally, one family (reported by the mother and her partner) indicated that their relatives have had COVID-19, although no hospitalization was required to treat the disease.

### 3.2. Descriptive Results

Considering the mean level of reported parents’ stress throughout the assessment month (corresponding to 15 measurements made every 2 days), we observed that the stress level tends to decrease slightly (between days 1 to 11). However, from day 12 on the stress level presents an oscillating pattern. When exposure to conflict and co-parenting support is considered, they show a similar pattern of results. Additionally, correlations indicate that—at most of the measurement times—parenting stress was related significatively and positively to exposure to conflict. In contrast, relationships between parents’ stress and co-parenting support, although they were inverse in nature, reached statistical significance in only 8 of the 15 measurement moments. Finally, a negative relationship between exposure to conflict and co-parenting support was significant at most of the measurement time points (Table 1).

Results show that mothers reported higher levels of previous depression than their partners. There were no differences between them in terms of the other time-invariant variables (see Table 2).

### 3.3. Trajectory of Change and Within-Person Fluctuation of Parental Stress

Model 1 indicates that, of the total variation of parents’ stress, 66% (ICC = 0.66) corresponds to between-person differences, and the remaining amount corresponds to within-person differences. Comparing Model 2 and Model 3, results show that the latter better accounts for the trajectory of parental stress (∆−2LL = 31.1, 1 df., *p* < 0.001). This model indicated that parents’ stress mean was 13.59 on the day of the first measurement and that their trajectory follows a decelerating negative function. Thus, the quadratic component (β = 0.03, *p* < 0.001) makes the negative linear effects of time (β = −0.44, *p* < 0.001) less negative as the days of confinement by COVID-19 go by (see Table 3).

Model 4 indicates that, considering the conflict and co-parenting support variables, the quadratic function continues to adequately describe the trajectory of stress reported by parents during the month in COVID-19 lockdown, measured by an EMA design. Additionally, the exposure to conflict is positively related to the level of parents’ reported stress, but there is no relationship between the latter and co-parenting support. This result is statistically significant, considering both parameters that capture within- and between-subject variability. Thus, this model indicates that for every one unit more exposure to conflict than usual (relative to the parent’s mean), that specific day’s level of stress is expected to be higher by 0.57 points. (*p* < 0.001), and that for every one-unit higher person-mean exposure to conflict, the mean number stress indicators reported across days is expected to be higher by 0.92 points (*p* < 0.001).

### 3.4. Predictors of Parent’s Stress Trajectory

**Figure 2:**
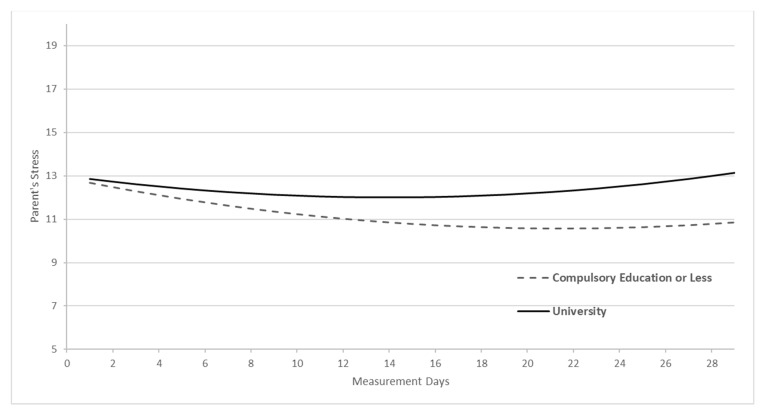
Parents’ educational level as predictor of parental stress trajectory.

Model 7 (see Table 4) shows the impact of education level, income, and number of children on participants’ stress trajectory during 29 days in the first full COVID-19 lockdown. The results indicate that neither the number of children nor the level of income were associated with the trajectory of participants’ stress level, both when considering the stress reported on the first day of the EMA measurement and throughout the month that this measurement was conducted. In contrast, the parents’ education level was related to parents’ reported stress. Specifically, there was an interaction between parents’ educational level and the lineal rate of change (β = 0.15, *p* < 0.05). This means that when parents have a university education, every other day the linear decrease of participants’ stress becomes less pronounced throughout the assessment month compared to parents with less education (See Figure 2).

With respect to control variables, both personality dimensions were positively related to parents’ stress (dependency and self-criticism). Thus, the higher the level of dependency or self-criticism, the higher the level of stress reported by parents.

## 4. Discussion

This study followed cohabiting parents of preschool children during a 30-day, COVID-19 lockdown period in Chile. Our results describe the trajectories and some of the factors that contributed to perceived parental stress during this period. In particular, we analyzed the relationship between stress and socioeconomic status/education, number of children, and co-parenting, finding mixed results in terms of our hypotheses.

### 4.1. Levels of Education and/or Income

Results did not support our first hypothesis that parents with higher levels of education and/or income would present lower stress at day one of assessment and a more adaptive trajectory of stress (i.e., no or less increase in stress levels during the follow-up period corresponding to one month at the beginning of the COVID-19 lockdown) compared to parents with lower educational level or income. The usual gap between individuals and families with more versus fewer resources (economic and/or educational) was not present, meaning that education and income did not function as a protective factor against COVID-19 related stress in this sample.

This could be due to the fact that people with fewer resources experienced and/or perceived less stress, or—to the contrary—people with more resources experienced more stress. In line with such a scenario, it may be that the government’s announcement of economic aid made on March 19 (see Figure 1) helped low-income families feel supported and thus less stressed by possible economic strains. Higher income households did not classify for economic aid and may not need this type of support, but given the lockdown they may have experienced the loss of other types of resources (such as paid help with child rearing or household chores) that entailed higher levels of uncertainty and stress.

In fact, the stress trajectory shown by more educated parents in our study could be related to evidence that suggests that higher educated parents have high academic expectations for their children and a higher sense of responsibility for their children’s education [67,68], even within low-income minority families [69]. It may have been difficult for these parents to meet this personal expectation because highly educated individuals are more likely to have jobs that required them to work from home during the lockdown [19], limiting the opportunity to monitor children’s home school activities. Accordingly, an Italian study that examined the consequences of the COVID-19 pandemic on job organization, found that people with lower educational levels had a lower risk of psychological distress than those with higher educational levels [70]. The authors argued that difficulties balancing working hours at home and daily care of children could have been particularly demanding for parents with executive roles who were required, more than others, to work from home. This fact combined with the higher concern about their children’s educational performance may have been an extra source of stress for more educated parents.

In Chile, for women from low SES, having to take care of others (i.e., children or the elderly) is one of the main reasons for not participating in the labor market. In fact, during the pandemic the female workforce was reduced by 21.5% compared to 2019. This impacted mostly low-skilled jobs such as housekeepers and waitresses, hitting the informal sector harder than it did the male workforce [71]. In this context, it is probable that mothers in our study with less education were unemployed during the lockdown, while more educated mothers continued working from home. Similarly, highly educated fathers may have also had to telework while their children were at home, thus facing the challenge of balancing work and supporting their children in school-related activities.

Moreover, it could be the case that families from higher SES relied on paid help for childcare or cleaning that became unavailable during lockdown [72], affecting the household organization for all family members.

Finally, it is important to remember that our study assessed the stress trajectory early on in the COVID-19 lockdown, which extended in Chile for several months during 2020, and its consequences continue to the present day. In this sense, our study captures an acute response to COVID-19-related challenges. It might be that resources associated with a higher education level may help families with long-term adaptation.

### 4.2. Number of Children per Household

With regard to the role of the number of children per household in perceived parental stress, contrary to our expectations, we found no significant results. Previous studies have suggested that having more children is associated with stress because it entails greater challenges (for example, dealing with relationships between siblings) and requires more time and energy devoted to caregiving activities [29]. Chilean studies have found similar results [30,73]. However, other studies argue that having very young children with low levels of autonomy could be more significant for parental stress than the number of children [74,75]. In Chile, Panesso and colleagues (2022) found that during the COVID-19 pandemic having to take care of children aged 0 to 4 years rather than the number of children was a predictor of parental burnout [76].

The families in our study had at least one child aged 5 to 6 years, and it may be that older children were able to entertain younger siblings and assist with some household chores, thus relieving their parents of some of these tasks. Accordingly, a recent study in Israel showed that during the COVID-19 pandemic, children were involved in some domestic and caregiving activities within the family context [77]. Similarly, a qualitative study in Chile found that mothers of children aged 6 to 12 years positively described an increase in their children’s autonomy associated with several activities including self-care (i.e., getting dressed, taking baths) and helping in daily household chores. This change was triggered by the significant reorganization of the usual family functioning, which both required more involvement from older children in daily tasks and provided the opportunity for parents to teach their children while engaging them in these activities [78]. Thus, it may be that in households with more children, the older ones were able to help adapt to the new conditions imposed by the COVID-19 pandemic by supporting parents in fulfilling their tasks instead of being a source of stress.

### 4.3. Co-Parenting Practices

When exploring the role of co-parenting practices in parental daily stress, we found that perceived support from the partner did not make a significant contribution; however, high co-parenting conflict was significantly associated with daily stress. An early study examining the asymmetric impact of positive and negative events suggests that most experiences trigger a mobilization response involving psychological and physiological aspects to cope with the event and minimize its potential negative impact. However, this mobilization response is greater for negative events, whereas positive and neutral experiences trigger more subtle mobilization responses [79]. Following this model, it may be possible that in our study the positive perception of a partner´s support and its associated response was not strong enough to impact the perception of stress, whereas the strong response related to the perception of conflict did impact perceived daily stress levels.

From another perspective, Kira and co-authors (2020) argue that the stress response is better understood from a macro-dynamic perspective that acknowledges the cumulative impact of diverse acute and chronic stressors, rather than the reaction to a single and more recent stressful event. Stressful events range from individual experiences to systemic stressors, such as natural disasters and pandemics. These stressors interact with each other and with individual characteristics across the lifetime, predisposing individuals to diverse resilience levels [80]. Due to the uncontrollable, unpredictable, and ongoing nature of the COVID-19 pandemic, parents were exposed to a chain of manifold stressful experiences, both acute and chronic, that pushed some individuals beyond their stress tolerance threshold [81]. In our study, conflict with a partner with whom there was no chance of maintaining distance due to confinement may have been the added stressor that triggered and/or maintained the stress response for some parents.

Our results should be interpreted with caution due to the characteristics of our sample, who were self-selected cohabiting parents of pre-school children. Thus, our results may not be generalizable to other groups. Additionally, although the measurement time frame we used falls within the ranges for an EMA study, it is possible that the follow-up was not long enough to capture the effect of our predictors on the trajectory of parental stress development. Future studies addressing parental stress in adverse circumstances could address this limitation by using longer follow-up times. Parents may have also experienced additional stressors (i.e., loss of an existing job or sources of practical support, etc.) during the assessment period that we did not anticipate and assess. Lastly, we assessed daily stress with self-report measures which, although appropriate for an EMA study, may not accurately represent objective stress levels. Furthermore, we focused on parents with at least one 5- to 6-year-old child, who likely helped with daily household tasks. Results could vary if studying the parents of younger children whose care is more demanding.

Future studies should investigate other possible sources of stress, such as physical health conditions and other mental health problems, as well as potential positive effects of the COVID-19 protective measures on psychological well-being during the pandemic.

Despite these limitations, the results of our study contribute to a better understanding of parental adaptation to uncommon sources of stress. We focused on acute adaptation during a specific moment in which parents had to handle COVID-19 related stressors, such as lockdown or the risk of getting sick with an unknown disease. Although the former was intended to reduce the virus’ spread, it was a novel situation that gave rise to unexpected consequences (i.e., the loss of a job or domestic support). This period imposed unprecedented demands that surpassed the resources related to traditional protective factors such as education level or income. Our study raises a note of caution to researchers and practitioners that protective factors need to be evaluated in context.

## 5. Conclusions

The COVID-19 pandemic was a source of significant stress for parents. Worries about the health of self and others, uncertainty about the virus and its broad consequences, and the universally imposed mobility restrictions demanded dramatic changes to the usual family functioning, forcing parents and children to adjust. Interestingly, our results contradict earlier evidence and show that expected protective factors (i.e., income and co-parental support) were unable to influence parents’ daily stress management during the initial weeks of lockdown. Moreover, parents with higher educational levels reported worse stress adaptation than less educated parents. Our results suggest that in unexpected and unusual situations, well-known protective risk factors may not work as such. Thus, it is relevant for both researchers and professionals to be aware of the possible limitations of our current knowledge, which may not be able to explain family dynamics in unusual circumstances. Future studies should keep investigating how parents adjust to stress to better understand the particular consequences that the COVID-19 pandemic had for parental emotional well-being.

## Figures and Tables

**Table 1:** Descriptive Results and Correlations of Time-varying Variables.

	Descriptive									Variable Correlations		
	Parents’ Stress			Exposure to Conflict			Co-Parenting Support			PS/EC	PS/CS	EC/CS
Days	M	DS	N	M	DS	N	M	DS	N	r	r	r
1	14.06	3.95	97	7.09	2.30	98	17.53	4.83	98	0.41 ***	−0.13	−0.37 ***
3	13.09	4.41	93	6.20	2.04	92	17.34	5.43	92	0.30 **	−0.32 **	−0.39 ***
5	12.83	4.47	78	5.81	1.73	79	16.34	5.83	80	0.32 **	−0.22 *	−0.28 *
7	11.63	3.93	87	6.03	2.47	88	16.42	6.11	89	0.14	−0.25 *	−0.19
9	11.19	3.81	86	5.63	1.44	84	17.34	5.61	87	0.34 **	−0.35 **	−0.07
11	11.18	3.86	79	5.52	1.18	77	17.29	5.88	79	0.40 ***	−0.15	−0.27 *
13	11.69	4.43	81	5.73	1.56	80	16.29	5.87	81	0.53 ***	−0.19	−0.24 *
15	11.28	4.36	86	5.59	1.54	86	16.56	5.68	82	0.37 ***	−0.11	−0.34 **
17	11.69	4.81	80	5.58	1.44	81	17.01	5.98	80	0.50 ***	−0.10	−0.31 **
19	11.53	4.44	80	5.59	1.36	79	15.96	6.16	76	0.40 ***	−0.28 *	−0.13
21	11.17	4.40	84	5.37	1.16	83	17.11	5.40	84	0.33 **	−0.22 *	−0.26 *
23	12.00	5.26	74	5.73	1.76	76	16.74	5.78	76	0.45 ***	−0.24 *	−0.37 ***
25	11.51	4.69	68	5.49	1.50	70	16.21	6.11	68	0.37 **	−0.12	−0.35 **
27	11.54	5.16	84	5.53	1.64	83	15.52	6.17	84	0.37 ***	−0.14	−0.26 *
29	11.55	4.83	77	5.46	1.27	80	16.22	6.20	79	0.31 **	−0.17	−0.22 *

Note. Correlations were estimated using pairwise deletion. PS = Parents’ stress, EC = Exposure to conflict, CS = Co-parenting support. * *p* < 0.05; ** *p* < 0.01; *** *p* < 0.001.

**Table 2:** Descriptive Results of Time-invariant Variables.

	Mothers’ Reports			Fathers’ Reports			*p*-Value Comparison
**Categorical Variables**	n (%)			n (%)			Chi-square
Number of Children	68 (100%)			63 (100%)			*p* = 0.667
1 Child	18 (27%)			13 (21%)			
2 Children	23 (34%)			21 (33%)			
3 or more Children	27 (40%)			29 (46%)			
Educational Level	66 (100%)			61 (100%)			*p* = 0.878
Compulsory Education or Less	24 (36%)			23 (38%)			
University	42 (64%)			38 (62%)			
Income	64 (100%)			58 (100%)			*p* = 0.607
Less than USD 380	13 (20%)			8 (14%)			
USD 380 to USD 1.300	42 (66%)			40 (69%)			
More than USD 1.300	9 (14%)			10 (17%)			
**Continuous variables**	*M*	*DS*	*N*	*M*	*DS*	*N*	*t-test*
Stressful events	1.88	1.31	68	1.76	1.09	63	*p* = 0.766
Parents’ previous depression	8.62	5.33	65	6.66	5.05	65	*p* = 0.017
Dependency	−0.95	0.70	62	−1.06	0.59	60	*p* = 0.173
Self-criticism	−0.87	0.85	62	−0.99	0.86	60	*p* = 0.136

**Table 3:** Fixed and Random Parameters of Linear Mixed Model of Parents’ stress.

	Model 1		Model 2		Model 3		Model 4	
	Estimator	SE	Estimator	SE	Estimator	SE	Estimator	SE
Model for the Means								
Intercept	12.28 ***	0.34	12.92 ***	0.33	13.59 ***	0.34	12.95 ***	0.32
Time ^a^			−0.10 **	0.03	−0.44 ***	0.06	−0.30 ***	0.06
Time × Time					0.03 ***	0.00	0.02 ***	0.00
Co-parental Support (within variability ^b^)							0.00	0.04
Exposure to Conflict (within variability ^b^)							0.57 ***	0.10
Co-parental Support (between variability ^c^)							−0.06	0.06
Exposure to Conflict (between variability ^d^)							0.92 ***	0.21
Model for the Variance								
Intercept	13.58 ***	1.87	11.71 ***	1.75	11.47 ***	1.71	9.35 ***	1.43
Time			0.07 ***	0.01	0.08 ***	0.01	0.06 ***	0.01
Covariance Intercept-Slope			−0.09	0.11	−0.11	0.12	−0.04	0.10
CS							0.03 *	0.01
Covariance CS-Intercept							−0.16	0.12
Covariance CS-Time							−0.01	0.01
EC							0.38 *	0.15
Covariance EC-Intercept							−0.05	0.32
Covariance EC-Time							−0.03	0.03
Covariance EC-CS							−0.08	0.05
Residual	6.95 ***	0.30	5.22 ***	0.23	5.02 ***	0.22	4.20 ***	0.20
REML Model Fit								
Number of parameters	3		6		7		18	
−2LL	6247.59		6060.99		6029.89		5761.15	
∆−2LL (∆ df.)	-		186.6 (3) ***		31.1 (1) ***		268.7 (9) ***	

Note. EC = Exposure to Conflict. CS = Co-parental Support. REML = Restricted Maximum Likelihood. ^a^ Time = 0 to 14. ^b^ Person mean-centered. ^c^ Person-mean of co-parental support across days − 16.6844. ^d^ Person-mean of exposure to conflict across days − 5.7832. * *p* < 0.05; ** *p* < 0.01; *** *p* < 0.001.

**Table 4:** Fixed and Random Parameters of Linear Mixed Model of Parents’ stress.

	Model 7	
	Estimator	SE
Model for the Means		
Intercept	12.68 ***	0.97
Time ^a^	−0.41 ***	0.11
Time × Tme	0.02 ***	0.00
Co-parental Support (within variability ^b^)	0.02	0.04
Exposure to Conflict (within variability ^b^)	0.57 ***	0.11
Co-parental Support (between variability ^c^)	−0.01	0.06
Exposure to Conflict (between variability ^d^)	0.92 ***	0.24
Mother	0.90	0.55
Parent Previous Depression ^e^	0.01	0.06
Stressful Events ^e^	−0.27	0.25
Dependency ^e^	1.36 **	0.48
Self-criticism ^e^	2.02 ***	0.39
Number of children ^f^		
Two children	−0.02	0.81
Three or more children	−0.18	0.81
Family income ^g^		
USD 380–USD 1300	−0.48	0.84
USD 380 vs. More than 1300	1.16	1.07
Parents Educational Level ^h^		
University	0.18	0.71
Time × One child vs. Two children	0.07	0.09
Time × One child vs. Three or more children	0.10	0.08
Time × USD 380–USD 1300	−0.07	0.09
Time × USD 380 vs. More than 1300	−0.13	0.11
Time × University	0.15 *	0.07
Model for the variance		
Random Intercept	6.47 ***	1.27
Random Time	0.06 ***	0.01
Covariance Intercept-Slope	−0.14	0.10
Random CS	0.02 *	0.01
Covariance CS-Intercept	−0.06	0.11
Covariance CS-Time	0.00	0.01
Random EC	0.26 *	0.13
Covariance EC-Intercept	−0.20	0.29
Covariance EC-Time	−0.01	0.03
Covariance EC-CS	−0.06	0.04
Residual	4.29 ***	0.22
REML Model Fit		
Number of parameters	33	
−2LL	4751.62	

Note. EC = Exposure to Conflict. CS = Co-parental Support. REML = Restricted Maximum Likelihood. ^a^ Time = 0 to 14. ^b^ Person-mean centered. ^c^ Person-mean of co-parental support across days − 16.6844. ^d^ Person-mean of exposure to conflict across days − 5.7832. ^e^ Control variables were grand-mean centered: Parent Previous Depression = Parent Previous Depression − 7.1714; Stressful Events = Stressful Events − 1.79. ^f^ Reference category = One Child. ^g^ Reference category = Less than USD 380 ^h^ Reference category = Compulsory Education or Less. * *p* < 0.05; ** *p* < 0.01; *** *p* < 0.001.

## Data Availability

The data presented in this study are available on request from the corresponding author. The data are not publicly available due to our commitment to protect the participants’ personal information.

## Supplementary Materials

The following supporting information can be downloaded at: https://www.mdpi.com/article/10.3390/ijerph20116008/s1, Model 7 was re-estimated including a COVID-19 diagnosis in the parents and their families see Table S1: Fixed and Random Parameters of Linear Mixed Model of Parents’ stress (Model 7 re-estimated).
