# Supplementary material for: Trajectories of Parental Daily Stress: An Ecological Momentary Assessment Study during the COVID-19 Lockdown
Source: Int J Environ Res Public Health. 2023 May 31;20(11):6008. doi: 10.3390/ijerph20116008 (PMC10252560; doi:10.3390/ijerph20116008)
Supplement: Supplementary file 1 [file ijerph-20-06008-s001.zip › ijerph-2213747-supplementary.pdf]

Suplemmentary Material

We analizyed COVID-19 diagnosis data available at the end of EMA assessment. These results show that: Six mothers (10%) reported having had the diagnostic test for the COVID-19, but the diagnosis was confirmed only in two cases (3%). Also, eight fathers (13%) reported the diagnosis process, and three of them (5%) were COVID-19 confirmed cases. Additinally, four families (6%) indicated that him/her and/or his/her relatives have had COVID-19 and hospitalization was required to treating it, and 17 families (27%) reported an outpatient treatment of COVID-19.

Conceptualizing a current COVID-19 diagnosis within the family as a relevant stressor and based on above results, the final model (i.e., model 7) was re-estimated including a COVID-19 diagnostic in the parents and their families as dummy predictors. The results are presented in the following Table (Model 8) alongside the original analysis (Model 7). These results indicated that the added predictors were not associated with the stress reported by parents (i.e. non - significant parameters). In addition, there are no changes in direction and/or significance of the other predictors.

Table S1. Fixed and Random Parameters of Linear Mixed Model of Parents' stress (Model 7 re-estimated)

	Model 7		Model 8	
	Estimator	SE	Estimator	SE
	Model for the Means			
Intercept	12.68***	0.97	12.60***	1.67
Time[a]	−0.41***	0.11	−0.41***	0.11
Time×Time	0.02***	0.00	0.02***	0.00
Co-parental Support (within variability[b])	0.02	0.04	−0.01	0.06
Exposure to Conflict (within variability[b])	0.57***	0.11	0.91***	0.25
Co-parental Support (between variability[c])	−0.01	0.06	−0.01	0.06
Exposure to Conflict (between variability[d])	0.92***	0.24	0.57***	0.11
Mother	0.90	0.55	0.85	0.57
Parent Previous Depression[e]	0.01	0.06	0.02	0.06
Stressful Events[e]	−0.27	0.25	−0.29	0.26
Dependency[e]	1.36**	0.48	1.37**	0.50
Self-criticism[e]	2.02***	0.39	2.04***	0.41
**COVID-19 Diagnosis in Parents or/and their relatives[f]**				

**Yes, and hospitalization required**	-	-	**−0.37**	**1.21**
**Yes, hospitalization not required**	-	-	**0.34**	**0.79**
**COVID-19 Exam[f]**				
**Yes, I am COVID-19 confirmed case**	-	-	**0.22**	**1.77**
**Yes, but I did not have COVID-19**	-	-	**0.14**	**1.39**
Number of children[g]				
Two children	−0.02	0.81	−0.08	0.82
Three or more children	−0.18	0.81	−0.18	0.83
Family income[h]				
USD 380 - USD 1.300	−0.48	0.84	−0.52	0.87
USD 380 vs. More than 1.300	1.16	1.07	1.13	1.11
Parents Educational Level[i]				
University	0.18	0.71	0.16	0.73
Time × One child vs. Two children	0.07	0.09	0.07	0.09
Time × One child vs. Three or more children	0.10	0.08	0.10	0.08
Time × USD 380 - USD 1.300	−0.07	0.09	−0.07	0.09
Time × USD 380 vs. More than 1.300	−0.13	0.11	−0.13	0.11
Time × University	0.15*	0.07	0.15*	0.07
Model for the variance				
Random Intercept	6.47***	1.27	6.78***	1.34
Random Time	0.06***	0.01	0.06***	0.01
Covariance Intercept-Slope	−0.14	0.10	−0.13	0.11
Random CS	0.02*	0.01	0.02*	0.01
Covariance CS-Intercept	−0.06	0.11	−0.06	0.12
Covariance CS-Time	0.00	0.01	0.00	0.01
Random EC	0.26*	0.13	0.26*	0.13
Covariance EC-Intercept	−0.20	0.29	−0.23	0.31
Covariance EC-Time	−0.01	0.03	−0.01	0.03
Covariance EC-CS	−0.06	0.04	−0.06	0.05

Residual	4.29***	0.22	4.29***	0.22
REML Model Fit				
Number of parameters	33		37	
−2LL	4751.62		4743.27	

Note. EC= Exposure to Conflict. CS=Co-parental Support. REML= Restricted Maximum Likelihood. [a]Time= 0 to 14. [b]Person mean-centered. [c]Person-mean of co-parental support across days − 16.6844. [d]Person-mean of exposure to conflict across days − 5.7832. [e]Control variables were grand-mean centered: Parent Previous Depression = Parent Previous Depression − 7.1714; Stressful Events = Stressful Events − 1.79. [f]Reference category= No. [g]Reference category= One-Child. [h]Reference category= Less than USD 380; [i]Reference category= Compulsory Education or Less.
$^{*}p < 0.05$; $^{**}p < 0.01$; $^{***}p < 0.001$.
